# A Critical History of Chromic Myeloid Leukemia

**DOI:** 10.4084/MJHID.2014.010

**Published:** 2014-01-02

**Authors:** Michele Baccarani, Fabrizio Pane

**Affiliations:** 1 University of Bologna and GIMEMA, Italy; 2 University of Naples “Federico II” and CEINGE Biotecnologie Avanzate, Naples, Italy

The history of chronic myeloid leukemia has marked one of the most remarkable and exciting success of modern medicine, transforming an almost always fatal cancer in a chronic conditions that can be medically controlled, and opening space and hope for a cure in an increasing number of patients.^1^–^6^ The discovery of the molecular bases of CML and of a class of agents, the tyrosine kinase inhibitors (TKIs), targeting the molecule that causes the leukemic transformation of hemopoietic stem cells, has provided a strongly positive support to the concept that cancer treatment must be based on the knowledge of cancer biologic characteristics and on the development of agents targeting specifically these characteristics. Until this concept remained a chimera, for many years, the treatment of cancer was necessarily based on cancer destruction exploiting the subtle differences in sensitivity among normal and cancer cells. This policy, a policy of “last cell kill”, gave positive results in many cancers. Examples, in hematology, are lymphomas, particularly Hodgkin’s lymphoma, and acute leukemia, particularly acute lymphoblastic leukemia in children. TKIs in CML as well as transretinoic acid in acute promyelocytic leukemia have at last shown that the concept of cancer-specific treatment is no longer a chimera and can be applied to clinical practice, in an increasing number of hematologic and non-hematologic malignancies.

The way was neither easy nor rapid. CML was described in the 19^th^ century and was managed with radiation and chemotherapy until the end of the 20^th^ century, until the introduction and the wide application of allogeneic stem cell transplantation (SCT) and of recombinant Interferon-alfa (rIFN ).^1^–^6^ SCT was in a sense the sublimation of the last cell kill theory, based on stem cell kill by radiation and alkylating agents and on residual stem cell control by the transplanted immune system. SCT is still the most important curative treatment of CML, but in spite of progress, the mortality and the morbidity are still important and limiting the application.^6^–^8^ As a matter of fact, SCT does not fit the concept of cancer specific treatment. rIFN was a great success, and an unexpected one, but also the benefit of rIFN was limited, because less than 50% of patients had a significant response, and side-effects were remarkable.^1^,^5^,^6^,^9^ Again, rIFN was not specifically directed against Ph+ cells, and it is intriguing to admit that after many years its mechanism of action is poorly known. After radiation and conventional chemotherapy, after allogeneic SCT, after rIFN, the true breakthrough was made by imatinib, the first of a class of small molecules inhibiting the TK.^10^ Soon after imatinib, other TKIs were rapidly developed and tested, and other four TKIs are now available, though not yet in all countries, and with different indications, dasatinib, nilotinib, bosutinib, and ponatinib.^11^–^15^ These compounds are similar and inhibit very effectively the kinase activity of BCR-ABL1, with a good therapeutic response, but are not identical. There are differences, in metabolism, pharmacokinetic and pharmacodynamics, there are differences in the ability of inhibiting BCR-ABL1 and particularly the mutated forms of BCR-ABL1, and there are differences in the inhibition of other different TK, leading to some differences in efficacy and to some differences in safety and side-effects. The major effect of the availability of more TKIs has been the relief of patients from dependence on only one drug, a condition that left too many patients at risk of dying of leukemia, and that was a cause of fear also in the responding patients. No more than 10 years ago, the big question was how long will the response last, how long will survival be.^1^ The early recognition of the development of TKIs-resistant mutations was a cause of fear and anxiety.^1^,^16^ We now know that with imatinib the responses are durable, that the risk of developing mutations decreases with time, that the majority of the patients who fail imatinib or are intolerant of imatinib can be rescue with other TKIs, and that a stable and deep response to imatinib may herald a treatment-free remission, a kind of clinical cure.^4^,^6^,^17^,^18^ However, the area of TKI and CML is still marked by a strong competition among drugs and companies, a competition that has comprehensible commercial bases, but has sound medical, ethical, and social consequences.^19^ Overall, the availability of more TKIs provides an unprecedented opportunity of designing the treatment according to disease and patient, that is to say of optimizing treatment, a goal that has not yet been reached because we must still learn how to do the best and proper use of these drugs. Many current treatment recommendations are based on efficacy, and the evaluation of efficacy is based sometimes too much on the so-called early surrogate markers of the outcome and sometimes too few on the outcomes, that are progression-free survival, overall survival, and treatment-free survival.^6^ Since the efficacy is high, it is necessary to take into consideration more and more also the safety and the tolerability, that affects so much the quality of life, and also the compliance, that in turn is important for the efficacy.^20^–^22^ It is true that the TKIs are targeted against BCR-ABL1 and are much less toxic and much better tolerated than most antileukemic and anticancer agents. But even TKIs are not completely specific, can cause important, and even life-threatening, clinical complications, as well as minor chronic side-effects that in the long term can become hard to tolerate.^20^–^26^ A careful monitoring of efficacy is necessary, based primarily on quantitative molecular testing and in case of failure also on mutational analysis, but also on cytogenetics, whenever molecular monitoring is not available or is not standardized according to the international scale.^6^,^27^,^28^ Monitoring is expensive, but the cost of careful monitoring is only a small fraction of the cost of treatment, and allows to make a proper use of any drug, from the clinical and also the financial point of view. However, monitoring the efficacy is not sufficient. Monitoring must be coupled with a careful attention to the patient, the comorbidities, the age, the complaints, the style of life, and ultimately the will of the patient.^20^,^26^–^29^ There is still a long and dedicated way to success. This issue of the Mediterranean Journal of Hematology and Infectious Disease covers some of the most important topics on the treatment of CML, to help health care professionals to walk along this way and to improve the management of CML patients.
